# Mitogenomics reveals extremely low genetic diversity in the endangered Jilin clawed salamander: Implications for its conservation

**DOI:** 10.1002/ece3.11132

**Published:** 2024-03-18

**Authors:** Yu Zhou, Ningkun Li, Hongjun Zhou, Ruoyan Zhou, Shuyan Cui, Guo Zheng

**Affiliations:** ^1^ College of Life Sciences Shenyang Normal University Shenyang China; ^2^ College of Life Science Northwest A&F University Yangling Shaanxi China

**Keywords:** historical demography, mitochondrial genome, nucleotide diversity, *Onychodactylus zhangyapingi*, species distribution modeling

## Abstract

The Jilin clawed salamander (*Onychodactylus zhangyapingi*) is an endemic, endangered, and level‐two protected amphibian species of China. In the context of serious threats to amphibians worldwide, conservation studies of this endangered species are urgently needed. In this study, mitogenomic conservation genetics and species distribution modeling analyses were performed for *O. zhangyapingi*. Sixty‐three samples were collected from nine different locations, and the complete mitochondrial genome was sequenced. Population genetic analyses revealed that *O. zhangyapingi* exhibits only one genetic structure with extremely low nucleotide diversity. Late Pleistocene climate cooling may have led to a reduction in effective population size and extremely low mitogenomic nucleotide diversity in this salamander, and the subsequent temperature increase (~20 kya to present) provided the opportunity for rapid population growth. The continuous highly suitable region for *O. zhangyapingi* is only approximately 3000 km^2^ on the southeastern boundary of Jilin Province, China. Fortunately, there are three large forested national nature reserves within the distribution of *O. zhangyapingi* that can effectively protect endangered species. Our findings suggest that *O. zhangyapingi* is a vulnerable species with a narrow distribution and extremely low genetic diversity, and we should pay more attention to the conservation management of this species.

## Graphical abstract

Mitogenomic conservation genetics of the endangered Jilin clawed salamander was performed in this study. Our findings showed that late Pleistocene climate cooling may have led to a reduction in the effective population size and extremely low mitogenomic genetic diversity of the Jilin clawed salamander. We suggest that *Onychodactylus zhangyapingi* is a vulnerable species and we should pay more attention to its conservation management.

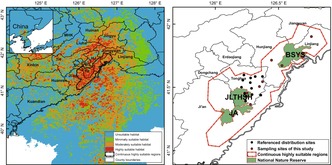

## 1 INTRODUCTION

Amphibians comprise a substantial, poorly understood, and highly endangered class of animals within vertebrates (Beebee, 2005; Luedtke et al., 2023; Shaffer et al., 2015). The second Global Amphibian Assessment revealed that 41% of the 8011 evaluated amphibian species are globally threatened (Luedtke et al., 2023). Climate change and habitat loss have become the two main factors threatening Amphibia over the past 20 years (Luedtke et al., 2023). The IUCN Red List Index shows that salamanders are the most strongly deteriorating amphibian group, and 58% of the evaluated salamander species fall into the threatened categories (extinct, extinct in the wild, critically endangered, endangered, vulnerable, or near threatened) (IUCN, 2023). China is rich in amphibian biodiversity, but these species are highly threatened, especially salamanders (Xie et al., 2007). The recently published endangered species on China's Red List of Biodiversity: Vertebrates lists 70 salamander species in China that are threatened, accounting for 72% of the 97 salamander species in China (AmphibiaChina, 2024). Therefore, comprehensive studies are needed on each threatened amphibian species as the basics of conservation management.

The Northeast Asian endemic genus *Onychodactylus* (Frost, 2023), which belongs to the family Hynobiidae, contains species that differ from other Hynobiidae species; in particular, they live in streams (Kuzmin, 1995), lack lungs, and have claws in larval and adult forms (Dunn, 1923). Within the genus *Onychodactylus*, the Jilin clawed salamander (*Onychodactylus zhangyapingi*) is known to be distributed narrowly in the Changbai Mountains of southeastern Jilin Province, China. The Jilin clawed salamander is a species endemic to China and is listed as an endangered species on China's Red List of Biodiversity: Vertebrates released by the Ministry of Ecology and Environment and the Chinese Academy of Sciences (https://www.mee.gov.cn) and level‐two protection species to the list of state‐protected wildlife coreleased by the National Forestry and Grassland Administration and the Ministry of Agriculture and Rural Affairs (https://www.gov.cn). For the endangered and mysterious *O. zhangyapingi*, many biological studies, such as studies on its life history, reproductive behavior, population genetics and even detailed distribution investigations, are lacking. This species comes out of hibernation annually beginning in early April and subsequently breeds from May to June (AmphibiaChina, 2024). They rest and hide under stones in clear streams during the daytime and nocturnally active (Poyarkov et al., 2012). To date, only four areas have been reported in the published literature with accompanying morphologically consistent images or homologous genetic data, including Heisonggou environs and nearby regions; Linjiang County, China (Poyarkov et al., 2012), Wunvfeng National Forest Park, Ji'an City, China (Luan et al., 2018); and Shihu Town and Laoling, Tonghua City, China (Luan et al., 2018).

Rare or endemic endangered species play important roles in ecosystems and are always important targets for conservation efforts (Frankham et al., 2002). The majority of endangered species that occupy narrow distribution ranges have low genetic diversity at the species level (Gong et al., 2010). Low genetic diversity can reduce the reproductive fitness and evolutionary potential of species and, ultimately, lead to extinction (Frankham et al., 2002; Spielman et al., 2004; Van Dyke, 2008). Therefore, for conservation management of endangered species, a distribution range investigation and population genetic analysis need to be performed. The Jilin clawed salamander is still needed to determine its current distribution and genetic status. In this study, we comprehensively collected specimens of *O. zhangyapingi* from nine locations throughout the Changbai Mountains on the southeastern border of Jilin Province, China, including Ji'an city, Tonghua County, Hunjiang District, and Linjiang city. The complete mitochondrial genomes were assembled for population genetic and historical demographic analyses. Additionally, species distribution models (SDMs) were generated to predict suitable areas for the Jilin clawed salamander. Conservation suggestions were subsequently provided based on the results of these analyses.

## 2 MATERIALS AND METHODS

### 2.1 Sampling, laboratory procedures, and sequencing

**FIGURE 1:**
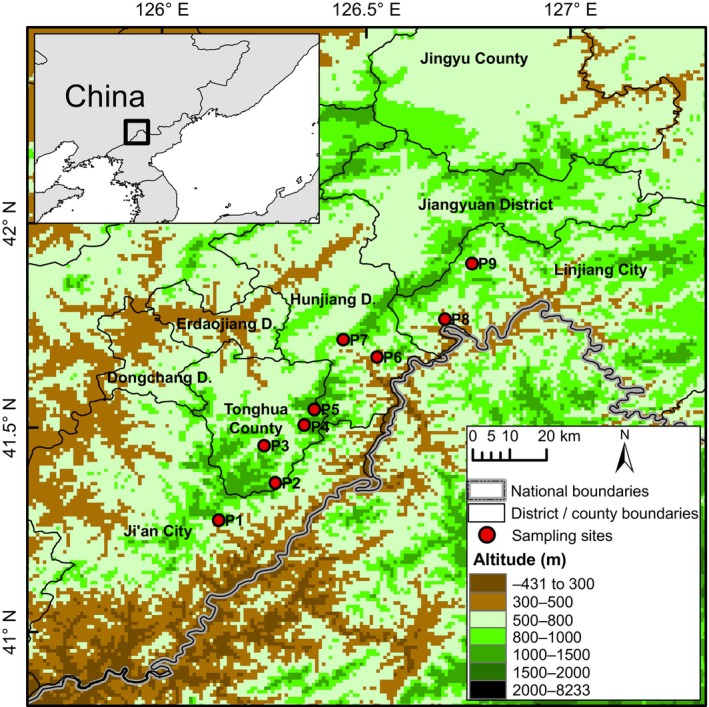
Locations of the 63 *Onychodactylus zhangyapingi* samples collected for this study.

Sixty‐three samples from nine locations were collected for our study during June 2023 (Table S1 and Figure 1). For each sample, only back toe‐tip (~1 mm^2^) tissues were cut off, preserved in absolute ethanol, and stored in sterile tubes. The living individuals were released immediately after the wounds were treated with antiseptic agents. Genomic DNA was extracted from the toe tip tissues using the TIANcombi DNA Lyse & Det PCR Kit (Beijing, China).

The mitogenome DNA pool of each specimen was prepared according to the parallel tagged amplicon sequencing method described by Feng et al. (2016). The mitochondrial genomes of the 63 Jilin clawed salamanders were amplified by two modified long‐PCR primers according to Zhou et al. (2021). These amplicon pools were purified using a TIANGEN universal DNA purification kit (Beijing, China). The DNA concentrations of the purified amplicon pools were measured using a Qubit 4 spectrophotometer. For each specimen, 2 μg of DNA from the cleaned amplicon pool was fragmented using NEBNext dsDNA fragmentase (NEB). The fragmented DNAs were purified and then blunt‐end‐repaired using T4 DNA polymerase (Takara, Inc., Dalian, China) and T4 DNA polynucleotide kinase (Takara Inc., Dalian, China). The blunt‐end‐repaired products were purified, and A‐tailing was subsequently performed using Premix Ex Taq (Takara Inc., Dalian, China).

For each species pool, species‐specific barcode linkers (with a minimum substitution distance ≥2 between the barcode linkers) were ligated to both ends of the fragments via TA ligation by using T4 DNA ligase (Takara, Inc., Dalian, China). Finally, the 63 mito‐DNA libraries were pooled directly. Finally, the pooled DNA was sequenced by Sangon Biotech (Shanghai, China) on an Illumina HiSeq 2500 sequencer. Approximately 18 GB of Illumina HiSeq paired‐end 150‐bp clean reads (filtering low‐quality data) were obtained.

### 2.2 Read sorting and assembly

Individual‐specific FASTQ sequence IDs were bioinformatically sorted with a custom Python script modified by Feng et al. (2016). The paired‐end FASTQ files were extracted by BBMap (Bushnell, 2014) based on the individual‐specific sequence IDs. Barcode linkers were removed using Cutadapt v1.8.2 (Martin, 2011). The mitogenomes of each individual were de novo assembled using NOVOPlasty (Dierckxsens et al., 2017) with a partial mitochondrial COX1 sequence from *O. zhangyapingi* (GenBank: JX158111) (Poyarkov et al., 2012) as the seed sequence. The raw read data for each individual are downloadable from the NCBI Sequence Read Archive (SRA) repository (accession SRS19446836–SRS19446898).

### 2.3 Sequence analyses

The complete mitogenomes were annotated using MITOS (Bernt et al., 2013). Nucleotide sequences were aligned using MAFFT version 5.0 (Katoh et al., 2005) with default parameters. Nucleotide diversity in each population and across all samples was calculated using DnaSP software version 6.12.03 (Rozas et al., 2017). Arlequin3.5 (Excoffier & Lischer, 2010) was used for molecular variance (AMOVA) (Excoffier et al., 1992), and significance was assessed based on 10,000 permutations.

### 2.4 Genetic clustering analysis

STRUCTURE v.2.3.4 (Pritchard et al., 2000) was used to determine the optimal number of genetic clusters (*K*) and to assign individuals to these genetic clusters. STRUCTURE calculations were conducted based on 100,000 burn‐ins and 1 million additional Markov Chain Monte Carlo (MCMC) chains. The *K*‐values were set from 1 to 10, with 10 replicates. The Puechmaille method (Puechmaille, 2016) was used to determine the optimum *K* as implemented on the STRUCTUR ESELECTOR (Li & Liu, 2017).

### 2.5 Historical demography

The demographic history of each population and all *O. zhangyapingi* samples were determined by means of neutrality tests and mismatch distributions in Arlequin (Excoffier & Lischer, 2010). Fu's *F*_s_ test (Fu, 1997) and Tajima's *D* test (Tajima, 1989) were applied to test whether the populations evolved under neutrality. Mismatch distributions (Harpending, 1994) were constructed using the sudden expansion model of Schneider and Excoffier (1999) with 10,000 bootstrap replicates, and the validity of the sudden expansion assumption was determined using sum of squares differences (SSDs) and Harpending's raggedness index (Hri) (Harpending, 1994).

BEAST v2.7.6 (Bouckaert et al., 2014) was used to perform Bayesian skyline plot (BSP) analysis (Drummond et al., 2005) to estimate the change in effective population size over time and the time to the most recent common ancestor (tMRCA). Three lognormal distributed calibrations, as reported by Zhang et al. (2006), were imposed on the BSP analysis. The root age was 110.7 (95% CI: 106.4, 114.9); the ancestor of *Hynobius*, *Salamandrella*, and *Batrachuperus* was 52.5 (95% CI: 50, 54.9); and the ancestor of *Ranodon*, *Hynobius*, *Salamandrella*, and *Batrachuperus* was 62.5 (95% CI: 59.7, 65.5). The four additional mitogenome sequences from GenBank were used to represent the genera *Ranodon*, *Hynobius*, *Salamandrella*, and *Batrachuperus*; DQ333818 of *Batrachuperus yenyuanensis*; JX508741 of *Salamandrella keyserlingii*; MN419307 of *Hynobius unisacculus*; and AJ419960 of *Ranodon sibiricus*. The best substitution model was determined using the bModelTest package (Bouckaert & Drummond, 2017) implemented in BEAST. Two independent analyses were performed using all the mitochondrial genome sequences available for each individual. MCMC was run with 10^9^ steps, sampling every 10,000 steps. The results of each run were visualized using TRACER 1.7 (Rambaut et al., 2018) to ensure that stationarity and convergence had been reached and that the effective sample size (ESS) was greater than 200.

### 2.6 Species distribution modeling

Species distribution models (SDMs) were predicted for *O. zhangyapingi* using the maximum entropy model implemented in Maxent v3.3.1 (Phillips et al., 2006). A total of 22 records were collected through field surveys (*n* = 9), from published literature records (*n* = 1; Xiong et al., 2016), and from the Global Biodiversity Information Facility (*n* = 12; https://doi.org/10.15468/dl.kb6rwe; GBIF.org 2024). Data for the 19 bioclimatic variables and elevation (at 30 arcseconds resolution) were downloaded from the WorldClim version 2.1 database as environmental data (Fick & Hijmans, 2017). Enhanced Vegetation Index (EVI) and Normalized Difference Vegetation Index (NDVI) in June 2022 were obtained from the NASA LPDAAC collection in the MODIS database (https://lpdaac.usgs.gov). The Forest/Non‐forest (FNF) map was downloaded from the Japan Aerospace Exploration Agency website: https://www.eorc.jaxa.jp/. The forest height data were downloaded from the Global Land Analysis & Discovery website: https://glad.umd.edu/dataset/gedi/ (Potapov et al., 2021). In SDM analysis, high multicollinearity among variables (Peterson & Nakazawa, 2008), regularization multipliers, and feature classes (Low et al., 2021) are the main factors that affect the prediction results (Radosavljevic & Anderson, 2014). To reduce this impact, we first used correlation analysis (Pearson's *r* > .85) (Dormann et al., 2013) in ENMTools (Warren et al., 2010) to minimize correlations between variables. Second, different regularization multipliers (0.5, 1, 1.5, 2, 2.5, 3, 3.5, and 4) and feature classes were assessed in R using the “kuenm” package (Cobos et al., 2019) to obtain the best combination of runs in Maxent (Phillips et al., 2006; Phillips & Dudík, 2008). Candidate model performance was evaluated based on significance (partial receiver operating curve (ROC), 100 iterations, and 50% of the data for bootstraps), omission rate (*E* = 5%), and model complexity (AICc). As a result, low multicollinearity (Figure S1) Annual Mean Temperature (BIO1), Mean Diurnal Range (BIO2), Isothermality (BIO3), Temperature Seasonality (BIO4), Max Temperature of Warmest Month (BIO5), Annual Precipitation (BIO12), Precipitation of Wettest Month (BIO13), Precipitation Seasonality (BIO15), Elevation (ELEV), Forest Height (FH), Forest/Non‐Forest (FNF), Normalized Difference Vegetation Index (NDVI), regularization multiplier 2, and feature class selected Linea features were used for SDM analysis.

## 3 RESULTS

### 3.1 Sequence information, genetic diversity, and genetic clustering

All individuals had target sequencing depths above 100× (Table S1). Among the 63 specimens, 61 mitogenomes were circularized, and two samples had only a partial mitogenome because of the failure of one part of the long‐PCR process (GenBank accession numbers: OR789810–OR789872).

The aligned mitogenome data set for *O. zhangyapingi* (16,474 bp) yielded 42 haplotypes among 63 sequences. The nucleotide diversity of each sampling location and all the samples exhibited extremely low values (Table 1). The lowest nucleotide diversity was 0.00003 in P3, the highest nucleotide diversity was 0.00033 in P1, and the nucleotide diversity of all the samples was 0.00014.

**TABLE 1:** Summary statistics of the demographic analysis of *Onychodactylus zhangyapingi*.

SA	*N*	*N*_H_	Tajima's *D* (*p‐*value)	*F*_s_ (*p‐*value)	ND (*π*)	SSD (*p‐*value)	Hri (*p‐*value)
P1	8	7	−1.721 (.021)	−1.174 (.200)	0.00033	0.024 (.703)	0.029 (.962)
P2	10	7	−1.388 (.099)	−3.424 (.003)	0.00013	0.017 (.421)	0.110 (.376)
P3	4	3	−0.612 (.376)	−0.887 (.091)	0.00003	0.113 (.192)	0.572 (.443)
P4 and P5	6	6	−1.390 (.049)	−3.395 (.012)	0.00014	0.009 (.891)	0.071 (.825)
P6	8	5	−1.064 (.151)	−1.403 (.088)	0.00011	0.018 (.580)	0.117 (.507)
P7	9	7	−1.149 (.139)	−4.814 (.001)	0.00014	0.435 (.001)	0.045 (.948)
P8	8	7	−1.640 (.021)	−3.802 (.004)	0.00009	0.021 (.520)	0.106 (.458)
P9	10	7	−1.136 (.134)	−0.459 (.376)	0.00008	0.027 (.298)	0.092 (.537)
All	63	42	−2.684 (.000)	−26.188 (.000)	0.00014	0.001 (.858)	0.022 (.764)

Abbreviations: Hri, Harpending's raggedness index; *N*, number of individuals; ND, nucleotide diversity; *N*_H_, number of haplotypes; SA, sampling location; SSD, sum of squares difference or mismatch distribution.

AMOVA revealed that within‐population diversity accounted for 99.26% of the overall variation, with a significant *p‐*value (.027) (Table S2). The diversity *among populations* was negligible, with values as low as 0.74%.

The four estimators of the Puechmaille method (*MedMeaK*, *MaxMeaK*, *MedMedK*, and *MaxMedK*) identified *K* = 1 as the most likely number of genetic clusters (Figure S2).

### 3.2 Historical demography

The neutrality test values (Tajima's *D* and *F*_s_) of each sampling location and all specimens were negative, especially for all specimens with significant *p‐*values (Table 1). The neutrality test indicated a significant recent demographic expansion in *O. zhangyapingi*. In the mismatch distribution, the *p*‐values of sudden demographic expansion (SSD and Hri values) were not significant, and thus, the hypothesis of recent demographic expansion of this species was not rejected.

**FIGURE 2:**
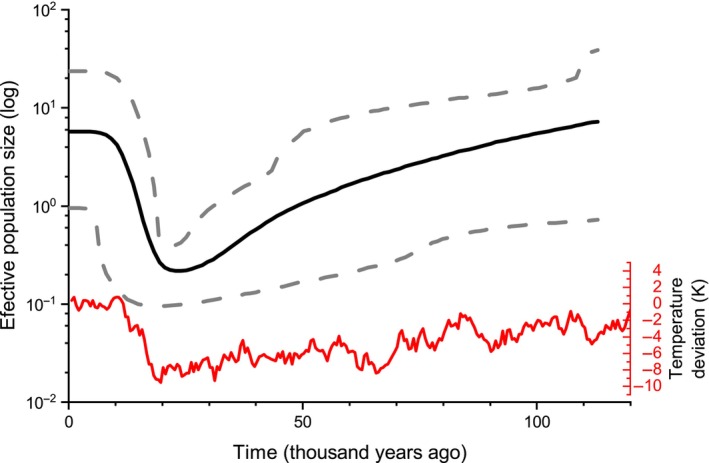
Bayesian skyline plot showing historical demographic trends in *Onychodactylus zhangyapingi*. The *x*‐axis shows the time before present in years, and the time goes backward from left to right. The *y*‐axis shows the effective population size on a log scale. The solid black lines represent the mean estimates, and the gray shaded areas represent the 95% confidence intervals. The red line represents the temperature deviation (Δ*T*site) from the mean of the last 100 kyr (Kawamura et al., 2007).

The BSP results suggested that the effective population size of *O. zhangyapingi* decreased approximately 110–20 thousand years ago (kya) and then rapidly increased approximately 20 kya until the present (Figure 2).

### 3.3 Species distribution modeling of *O. zhangyapingi*

The SDMs generated excellent predictions of occurrence locations under current climate scenarios. The AUC for *O. zhangyapingi* was 0.994. The jackknife test showed that the seven main contributing variables were BIO13 (18.9%), NDVI (17.6%), FNF (15.7%), BIO15 (13.6%), BIO2 (12.9%), BIO1 (9.5%), and ELEV (7.1%), with a total contribution of 95.3% (Table S3).

**FIGURE 3:**
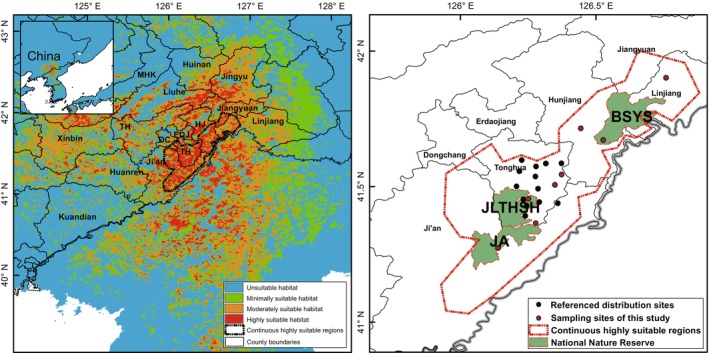
Predicted distribution of *Onychodactylus zhangyapingi* (left) and the internal distribution of national natural reserves (right).

The highly suitable regions for *O. zhangyapingi* were clustered in the Changbai Mountains of Ji'an city, Tonghua County and Hunjiang District, which are the southeastern boundaries of Jilin Province, China (Figure 3). Combined with our sampling sites, the continuous highly suitable region for *O. zhangyapingi* is in the Changbai Mountains of Ji'an city, Tonghua County, Hunjiang District, and Linjiang city on the southeastern boundary of Jilin Province, with an area of approximately 3000 km^2^ (Figure 3).

## 4 DISCUSSION

### 4.1 Mitogenome assembly

In this study, for the first time, we revealed the population genetics and distribution of the endangered species *O. zhangyapingi* based on mitogenome sequences. Sixty‐one complete mitogenomes and two partial mitogenomes were successfully assembled with high sequencing depth (Table S1). Although the mitogenome has limitations, such as limited sequence lengths, maternal inheritance, and no DNA recombination compared with nuclear molecular markers, it is also widely used for molecular ecology analyses, such as estimating genetic diversity, finding evolutionarily significant units and evaluating phylogeography (Galtier et al., 2009; Naik, 2017; Wan et al., 2004). Therefore, in this study, mitogenomic sequence information was successfully used to evaluate the genetic diversity, genetic clustering, and historical demography of *O. zhangyapingi*.

### 4.2 Single genetic cluster of Jilin clawed salamanders

One of the main steps in endangered species conservation genetics is to clarify their genetic structure (O'Brien, 1994). Genetic structure is considered a key factor in the short‐term evolution of a population (Ng et al., 2004). Knowledge of genetic structure can facilitate the elucidation of the makeup of an evolutionarily significant unit or subspecies of an endangered species and provide conservation and management strategies (Hu et al., 2020; O'Brien, 1994; Yan et al., 2018). For Jilin clawed salamanders, no different evolutionarily significant units or even significantly different clusters were found via STRUCTURE analysis. AMOVAs revealed that a significant amount (99.26%) of the overall variation occurred within populations (Table S2), which additionally supports a single genetic structure within *O. zhangyapingi*. The low genetic diversity and single genetic structure imply that *O. zhangyapingi* should be treated as a whole in terms of conservation.

### 4.3 Historical demography during the late Pleistocene

To respond to cyclical climatic changes in the Pleistocene, species could repetitively expand their ranges by an “expansion‐contraction” strategy during alternating glacial and interglacial periods (Provan & Bennett, 2008). The BSP analysis showed that the effective population size of *O. zhangyapingi* decreased from approximately 110–20 kya and then rapidly increased beginning at approximately 20 kya (Figure 2). Recent expansions of the effective population size were also confirmed by neutrality tests and mismatch distribution analyses (Table 1). The global temperature decreased from 130 kya to approximately 25 kya, which was the Last Glacial Maximum (Bassinot et al., 1994; Kawamura et al., 2007; Lisiecki & Raymo, 2005). The Last Glacial Maximum climate changes were positively related to the phylogenetic clustering of extant Chinese terrestrial vertebrates (Huang et al., 2023). In this study, the BSP showed almost the same variation trend as that of late Pleistocene climate change when compared with the late Pleistocene northern hemisphere temperature change reported by Kawamura et al. (2007) (Figure 2). Late Pleistocene climate change disastrously affected *O. zhangyapingi* by decreasing the effective population size and reducing genetic diversity. Notably, *O. zhangyapingi* did not adapt to late Pleistocene climate cooling. The Jilin clawed salamander is a long‐term stream‐adapted species with feeble movement abilities. During the climate cooling stages, *O. zhangyapingi* had difficulty migrating to the refuge because of their singular habitation. Therefore, late Pleistocene climate cooling may have had catastrophic impacts on *O. zhangyapingi* and led to a decrease in its distribution area and population size. The subsequent temperature increase (~20 kya to present) provided the opportunity for effective population size expansion. The same late Pleistocene demographic history was also observed for some other organisms (Liu et al., 2019; Ma et al., 2018; Miller et al., 2012; Wang et al., 2018).

### 4.4 Suitable distribution

The distribution of *O. zhangyapingi* was mainly affected by vegetation (NDVI and FNF), precipitation (BIO15 and BIO13), and temperature (BIO2 and BIO1), which suggested that *O. zhangyapingi* has adapted to climate and biological conditions in its current habitat. The Jilin clawed salamander is an endemic species of China. The SDM analysis also revealed that the continuous highly suitable habitat for *O. zhangyapingi* occurs in the Changbai Mountains of Ji'an city, Tonghua County, Hunjiang District, and Linjiang city on the southern border of Jilin Province. Our sampling sites were all in streams on one continuous mountain (Figure 1). Therefore, although there are many fragmented highly suitable regions around our defined continuous highly suitable habitat, such as in the northeastern Liaoning Province of China and northern Democratic People's Republic of Korea (Figure 3), whether *O. zhangyapingi* has inhabited these regions requires further investigation.

### 4.5 Implications for conservation

*O. zhangyapingi* and *O. zhaoermii* are the two endemic *Onychodactylus* species of China and have different protection levels. *O. zhaoermii* is a critically endangered species and level‐one protected species of China, and *O. zhangyapingi* is an endangered species and level‐two protected species of China. The current population of *O. zhangyapingi* may be very small. Each of the nine sampling lines within this study were on both sides of an approximately 500‐m‐long stream. Three investigators carefully turned over almost every stone, ultimately obtaining a total of 63 samples (average 7 individuals per site) with 25 subadult and 38 mature individuals, which were all included in this study. The SDM analysis revealed that the continuous highly suitable region for *O. zhangyapingi* is approximately 3000 km^2^, and the narrow distribution range meets the criteria for endangered animals. Our mitogenomic nucleotide diversity analysis revealed an extremely low value for *O. zhangyapingi* (0.00014 of all samples, Table 1), which is lower than the *mitogenomic nucleotide diversity* of *O. zhaoermii* (Zhou et al., 2021), a critically endangered clawed salamander of China. Reductions in genetic diversity can have serious implications for the evolutionary potential of a species (Frankham, 2005; Kahilainen et al., 2014). The extremely low nucleotide diversity implies that *O. zhangyapingi* is facing a more serious genetic diversity crisis than *O. zhaoermii*. Within the IUCN Red List of Threatened Species, *O. zhangyapingi* is data deficient. Due to the ultralow genetic diversity, narrow distribution and current small population, we suggest that *O. zhangyapingi* be given more attention regarding the IUCN Red List in the future.

Fortunately, within the approximately 3000 km^2^ of continuous highly suitable area for *O. zhangyapingi*, there are three large forested National Nature Reserves, the Ji'an National Nature Reserve, Jilin‐Tonghua‐Shihu National Nature Reserve, and Baishan‐Yuanshe National Nature Reserve. These national nature reserves can continuously protect habitats and all living organisms and provide hope for the future conservation of *O. zhangyapingi*.

## AUTHOR CONTRIBUTIONS

**Yu Zhou:** Conceptualization (equal); formal analysis (equal); investigation (equal); resources (equal); writing – original draft (equal); writing – review and editing (equal). **Ningkun Li:** Data curation (equal); investigation (equal); software (equal); writing – original draft (equal). **Hongjun Zhou:** Data curation (equal); investigation (equal); software (equal). **Ruoyan Zhou:** Investigation (equal); software (equal); visualization (equal). **Shuyan Cui:** Formal analysis (equal); writing – review and editing (equal). **Guo Zheng:** Conceptualization (equal); project administration (equal); writing – review and editing (equal).

## FUNDING INFORMATION

This study was supported financially by the Research Fund for the Liaoning Revitalization Talents Program (Grant No. XLYC2002083), the Liaoning Province Science and Technology Plan Project (Grant No. 2018103004), the Educational Commission of Liaoning Province of China (Grant No. LQN201904), and the Natural Science Foundation of Liaoning Province of China (Grant No. 2020‐BS‐148).

## CONFLICT OF INTEREST STATEMENT

The authors declare that they have no competing interests.

## Supporting information


Appendix S1

## Data Availability

The mitochondrial DNA sequences have been deposited in the National Centre for Biotechnology Information (NCBI) and the accession numbers are OR789810–OR789872. The Illumina reads are available in the NCBI Sequence Read Archive (SRA) repository (accession numbers SRS19446836–SRS19446898).
