# Supplementary material for: Mitogenomics reveals extremely low genetic diversity in the endangered Jilin clawed salamander: Implications for its conservation
Source: Ecol Evol. 2024 Mar 18;14(3):e11132. doi: 10.1002/ece3.11132 (PMC10948371; doi:10.1002/ece3.11132)
Supplement: Supplementary file 1 — Appendix S1 [file ECE3-14-e11132-s001.docx]

**Supplementary**

**Table S1** Information for the Jilin clawed salamanders included in this study.

| Voucher number | Locality | Population | Coverage depth |
| --- | --- | --- | --- |
| ZY-23062206119 | Wunvfeng National Forest Park, Ji'an City, China | P1 | 718 |
| ZY-23062206120 |  | P1 | 2410 |
| ZY-23062206121 |  | P1 | 11028 |
| ZY-23062206122 |  | P1 | 335 |
| ZY-23062206123 |  | P1 | 5559 |
| ZY-23062206125 |  | P1 | 10921 |
| ZY-23051606127 |  | P1 | 4135 |
| ZY-23051606128 |  | P1 | 586 |
| ZY-2306240699 | Shihu National Forest Park, Tonghua County, China | P2 |  |
| ZY-23062406100 |  | P2 | 4174 |
| ZY-23062406101 |  | P2 | 3225 |
| ZY-23062406102 |  | P2 | 2095 |
| ZY-23062406103 |  | P2 | 5963 |
| ZY-23051606096 |  | P2 | 2516 |
| ZY-23051606104 |  | P2 | 23029 |
| ZY-23051606105 |  | P2 | 10688 |
| ZY-23051606106 |  | P2 | 120 |
| ZY-23051606107 |  | P2 | 16606 |
| ZY-23062607134 | Yongan village, Tonghua County, China | P3 | 25155 |
| ZY-23062607135 |  | P3 | 6465 |
| ZY-23062607137 |  | P3 | 10124 |
| ZY-23062607139 |  | P3 | 53777 |
| ZY-23062607140 | Qidaogou, Tonghua County, China | P4 | 3524 |
| ZY-23062604151 |  | P4 | 9052 |
| ZY-23062604152 |  | P4 | 7035 |
| ZY-23062604153 |  | P4 | 8509 |
| ZY-2306260282 | Qidaogou, Tonghua County, China | P5 | 10966 |
| ZY-2306260283 |  | P5 | 14747 |
| ZY-23062505126 | Xiasandaogou, Hunjiang District, China | P6 | 12461 |
| ZY-23062505127 |  | P6 | 4706 |
| ZY-23062505128 |  | P6 | 7703 |
| ZY-23062505129 |  | P6 | 11094 |
| ZY-23062505130 |  | P6 | 6652 |
| ZY-23062505131 |  | P6 | 3619 |
| ZY-23062505132 |  | P6 | 2228 |
| ZY-23062505133 |  | P6 | 17010 |
| ZY-2306250372 | Qishierdaogou village, Hunjiang District, China | P7 | 7919 |
| ZY-2306250373 |  | P7 | 9011 |
| ZY-2306250374 |  | P7 | 51396 |
| ZY-2306250375 |  | P7 | 6345 |
| ZY-2306250376 |  | P7 | 2581 |
| ZY-2306250377 |  | P7 |  |
| ZY-2306250379 |  | P7 | 1267 |
| ZY-2306250380 |  | P7 | 1328 |
| ZY-2306250381 |  | P7 | 3798 |
| ZY-230519061901 | Jinyinxia, Linjiang City, China | P8 | 7527 |
| ZY-230519061902 |  | P8 | 4385 |
| ZY-230519061903 |  | P8 | 2995 |
| ZY-230519061904 |  | P8 | 6612 |
| ZY-230519061905 |  | P8 | 6289 |
| ZY-230519061906 |  | P8 | 2208 |
| ZY-230519061907 |  | P8 | 11178 |
| ZY-230519061908 |  | P8 | 13119 |
| ZY-2306280102 | Zhenzhumen village, Linjiang City, China | P9 | 23556 |
| ZY-2306280103 |  | P9 | 20017 |
| ZY-2306280104 |  | P9 | 16710 |
| ZY-2306280105 |  | P9 | 12929 |
| ZY-2306280106 |  | P9 | 16906 |
| ZY-2306280107 |  | P9 | 2017 |
| ZY-2306280108 |  | P9 | 6260 |
| ZY-2306280109 |  | P9 | 9213 |
| ZY-2306280110 |  | P9 | 23813 |
| ZY-2306280111 |  | P9 | 12091 |

**Table S2.** Results of AMOVA

| Source of variation | d.f. | Sum of squares | Percentage of variation | *P* value |
| --- | --- | --- | --- | --- |
| Among populations within groups | 7 | 13.049 | 0.74 | 0.02844 |
| Within populations | 53 | 93.508 | 99.26 | 0.0000 |

**Table S3.** Percent contribution and permutation importance of the environmental variables in the Maxent model.

| Variable | Description | Percent contribution | Permutation importance |
| --- | --- | --- | --- |
| BIO13 | Precipitation of Wettest Month | 18.9 | 4.2 |
| NDVI | Normalized Difference Vegetation Index | 17.6 | 3.3 |
| FNF | Forest/non-forest | 15.7 | 8.4 |
| BIO15 | Precipitation Seasonality | 13.6 | 1.6 |
| BIO2 | Mean Diurnal Range | 12.9 | 29.4 |
| BIO1 | Annual Mean Temperature | 9.5 | 33 |
| ELEV | Elevation | 7.1 | 13.9 |
| BIO3 | Isothermality (BIO2/BIO7) (* 100) | 3.5 | 6.3 |
| BIO5 | Max Temperature of Warmest Month | 1.1 | 0 |
| BIO12 | Annual Precipitation | 0 | 0 |
| BIO4 | Temperature Seasonality (standard deviation *100) | 0 | 0 |
| FH | Forest height | 0 | 0 |


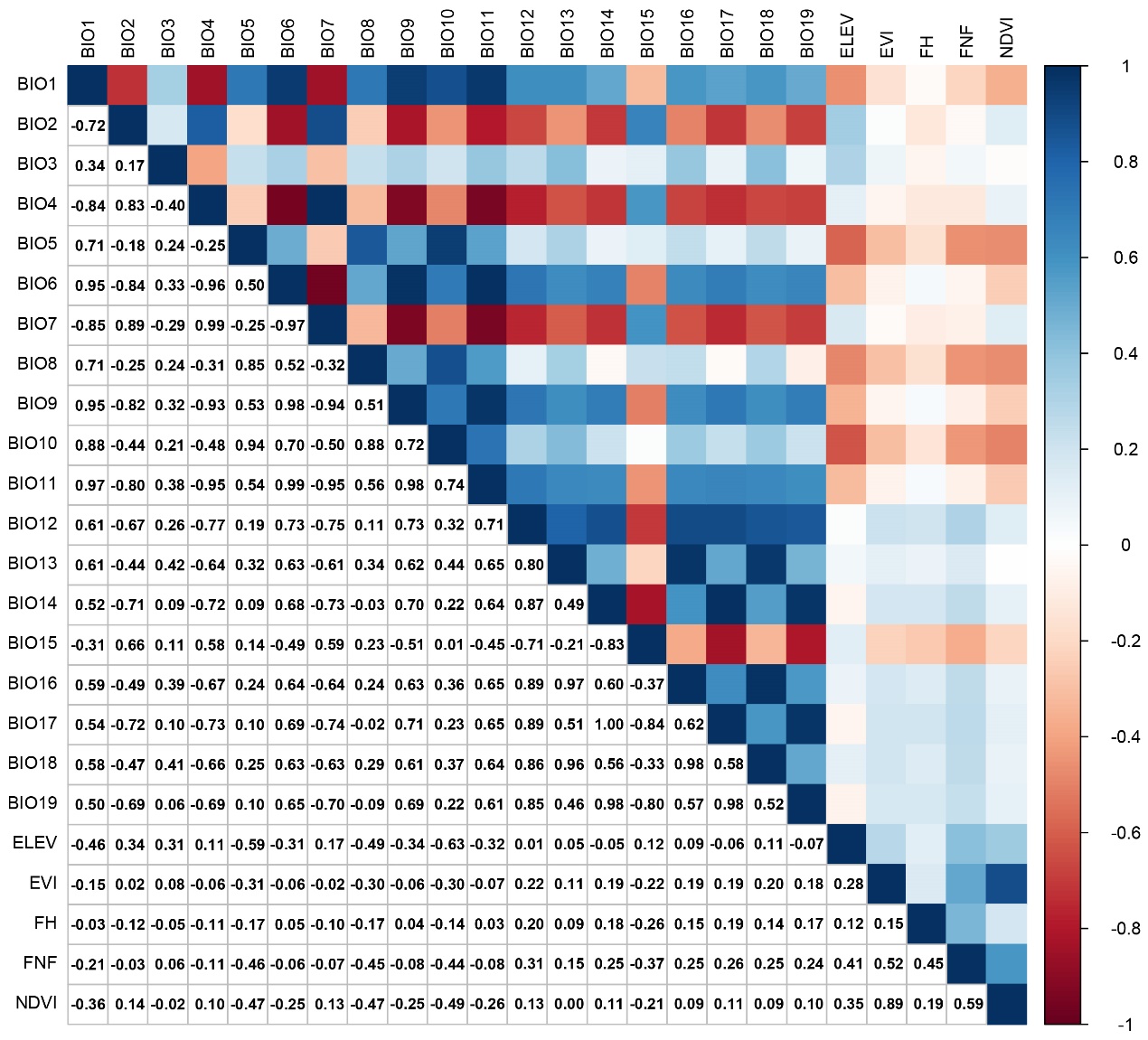


Figure S1 Pearson correlation matrix of 24 environmental variables and elevation.


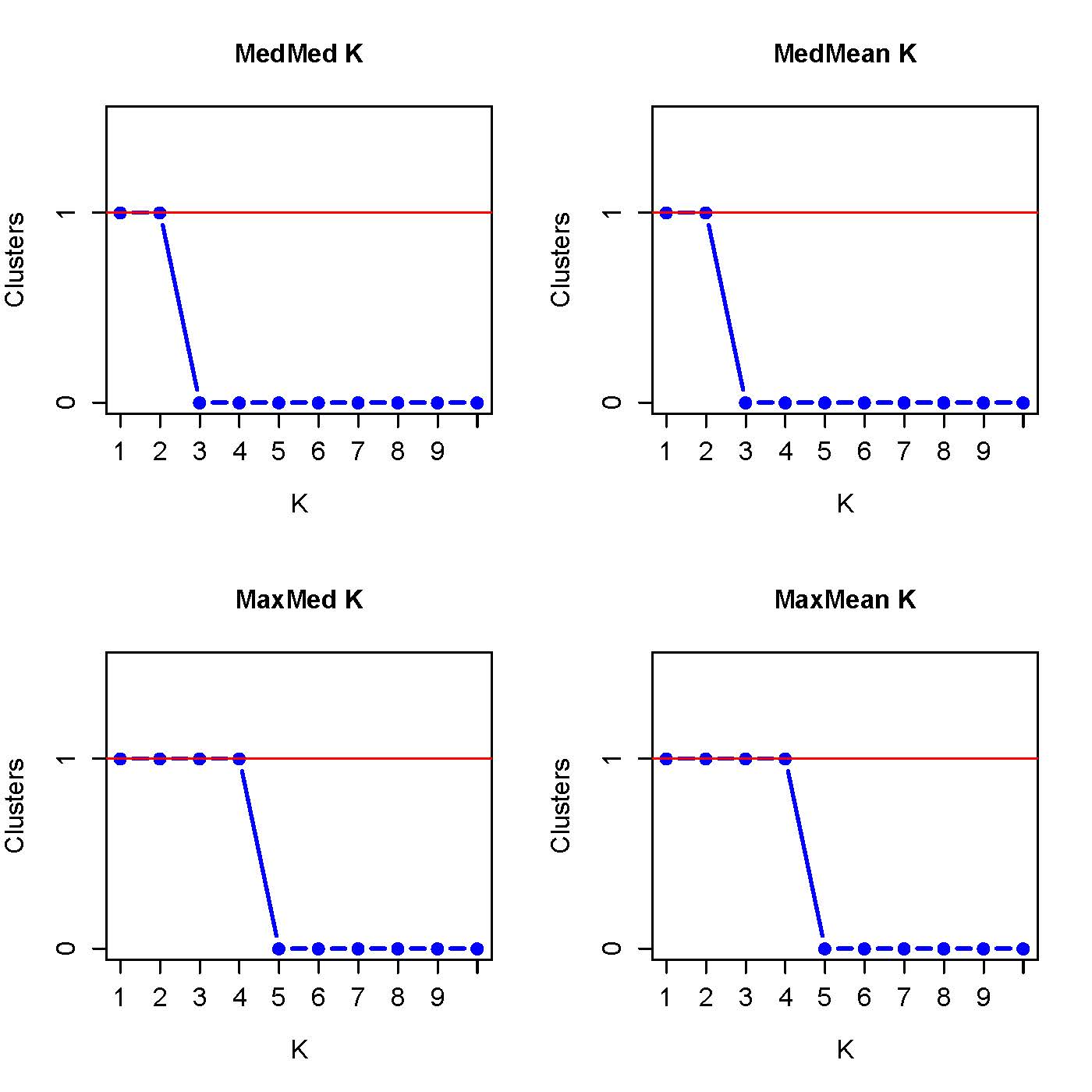


Figure S2 The four estimators of the Puechmaille method (*MedMeaK*, *MaxMeaK*, *MedMedK* and *MaxMedK*) for the Bayesian clustering (STRUCTURE) for *Onychodactylus zhangyapingi*.
